# Biotransformation of Major Ginsenoside Rb_1_ to Rd by *Dekkera anomala* YAE-1 from Mongolian Fermented Milk (Airag)

**DOI:** 10.4014/jmb.2004.04022

**Published:** 2020-08-14

**Authors:** Gereltuya Renchinkhand, Soo-Hyun Cho, Young W. Park, Gyu-Yong Song, Myoung Soo Nam

**Affiliations:** 1 Department of Animal Biosystem Science, College of Agriculture and Life Sciences, Chungnam National University, Daejeon 34134, Republic of Korea; 2 Department of Pharmacology Science, College of Pharmacology, Chungnam National University, Daejeon 34134, Republic of Korea; 3 Agricultural Research Station, Fort Valley State University, Fort Valley, GA 31030, USA and and Department of Food Science and Technology, University of Georgia, Athens, GA 30602, USA

**Keywords:** Fermented mare’s milk, airag, ginsenoside, β-glucosidase, *Dekkera anomala* YAE-1

## Abstract

*Dekkera anomala* YAE-1 strain separated from “airag” (Mongolian fermented mare’s milk) produces β-glucosidase, which can convert ginsenoside Rb_1_ from *Panax ginseng*. Ginseng-derived bioactive components such as ginsenoside Rb_1_ have various immunological and anticancer activities. Airag was collected from five different mare milk farms located near Ulaanbaatar, Mongolia. YAE-1 strains were isolated from airag to examine the hydrolytic activities of β-glucosidase on Korean *Panax ginseng* using an API ZYM kit. Supernatants of selected cultures having β-glucosidase activity were examined for hydrolysis of the major ginsenoside Rb_1_ at 40°C, pH 5.0. The YAE-1 strain was found to be nearly identical at 99.9% homology with *Dekkera anomala* DB-7B, and was thus named *Dekkera anomala* YAE-1. This strain exerted higher β–glucosidase activity than other enzymes. Reaction mixtures from *Dekkera anomala* YAE-1 showed great capacity for converting ginsenoside Rb_1_ to ginsenoside Rd. The β-glucosidase produced by *Dekkera anomala* YAE-1 was able to hydrolyze ginsenoside Rb_1_ and convert it to Rd during fermentation of the ginseng. The amount of ginsenoside Rd was highly increased from 0 to 1.404 mg/ml in fermented 20% ginseng root at 7 days.

## Introduction

In Korea, ginseng products are widely used as health functional foods and generally enjoy brisk sales. In 2018, health functional food sales totaled US$2.1 billion and red ginseng products accounted for 44%, or US$927 million in sales [1]. Biologically functional components found in most ginseng species include ginsenosides, polysaccharides, peptides, polyacetylenic alcohols, and fatty acids [2]. Ginsenoside Rd is the main hydrolyzed product of ginsenosides Rb_1_, Rb_2_, and Rc, which are components in more than 80% of ginseng roots [3, 4]. Among ginsenosides, the minor ginsenoside Rd has been reported to have important biological activities including immunosuppressive [5] and anti-inflammatory properties [6]. Ginsenosides Rk1 and Rg5, products of ginsenoside Rd, prevent wrinkle formation and the loss of collagen from epithelial cells [7]. Moreover, the anti-inflammatory properties of ginsenoside Rd inhibit iNOS and COX-2, which are known to play pivotal roles in the pathogenesis of acute and chronic inflammation. Ginsenoside Rd has therapeutic effects on inflammation and may therefore have applications in the treatment of inflammatory diseases [8]. Moreover, ginsenoside Rd prevents Pb-induced decrease in NSC proliferation by inhibiting microgliosis [9].

Airag is the local name for fermented mare’s milk in Mongolia. The traditional beverage is fermented by a co-culture of yeasts and lactic acid bacteria and is produced by churning mare’s milk in a leather sack with a wooden masher.

Airag does not use an inoculated starter and is instead fermented by mixing the lactic acid bacteria and yeast in mare’s milk. Therefore, the diversity of yeast and lactic acid bacteria is very high.

*Dekkera anomala* is one of several spoilage yeasts found in the genus *Dekkera*. *D. anomala* was differentiated from other species of the genus morphologically by the formation of blastese, single filamentous pseudohyphae, and physiologically by the fermentation of lactose [10].

The conversion of ginsenosides by LAB enzymes has great potential importance for human health due to biological, immunological and medicinal functions. We have hypothesized that the β-glucosidase activity of the *D. anomala* YAE-1 could be used to hydrolyze the ginsenoside Rb_1_. The objectives of this study are to separate and characterize *D. anomala* YAE-1 having β-glucosidase activity from airag, and to investigate the enzymatic capacity of *D. anomala* YAE-1 strain on the hydrolysis of a major ginsenoside (Rb_1_) in Korean ginseng.

## Materials and Methods

### Collection of Airag Samples

Airag was prepared according to the method [11]. Airag samples were collected from four different mare milk farms located in close proximity to Ulaanbaatar, Mongolia.

### Isolation of Strains with β-Glucosidase Activity from Airag

The experimental strains were isolated from airag, a Mongolian fermented milk product. Samples were plated according to the method [12-14]. Esculin-positive colonies were inoculated in pH 4.0 Yeast Malt broth (HiMedia Laboratories, USA), and sample preparation and β-glucosidase activity were determined by the procedure [12]. Conversion of ginsenosides was checked by TLC analysis [15].

### 18S Ribosomal RNA Gene Sequencing of the Strain YAE-1 with β-Glucosidase Activity

18S rRNA gene sequencing of selected strains was conducted according to the method [12]. PCR sequences were compared with those in the NCBI database using BLAST. The primers used were ITS1: 5'-TCCGTAGGTGAACCTGCGG-3' and ITS4: 5'-TCCTCCGCTTATTGATATGC-3'.

### Effect of Temperature and pH on Growth of *D. anomala* YAE-1

To determine the optimum pH (3.0-7.0) and temperature (20-40°C) for growth of strain YAE-1, 3% (v/v) of the yeast strain was inoculated in YM broth for 72 h in an incubator, and the specimen was checked for viable cell count.

### Enzyme Activity by API ZYM Kit

Enzyme activity was detected using the API ZYM Kit (BioMérieux, Mercy I’Etoile, France) in accordance with the manufacturer's instructions. Selected colonies from the surface agar plates were suspended in medium (0.085% NaCl, Ref. 20070, BioMérieux,) to a turbidity adjusted to a McFarland No. 5.0-6.0 standard (BioMérieux). Each microcupule of the API ZYM gallery containing 19 dehydrated chromogenic enzyme substrates was inoculated with 50 μl of the suspension, and the strip was incubated 4 h at 37°C. (BioMérieux).

### Analysis of Carbohydrate Utilization

Carbohydrate fermentation tests were performed according to the method [11]. Selected colonies from the surface agar plates were suspended in medium (0.085% NaCl) to a turbidity adjusting to a McFarland No. 2.0 standard (BioMérieux) and inoculated into API C medium. Each microcupule of the API 20C AUX kit containing 19 carbohydrates with pH detector enzyme substrates was inoculated with 100 μl of the suspension. The first microcupules were used as a negative control. The strips were incubated 48 and 72 h at 29 ± 2°C.

### β-Glucosidase Activity of *D. anomala* YAE-1 Supernatant 

β-Glucosidase activity of the supernatant was determined by the rate of hydrolysis of 5 mM p-nitrophenyl-β-D-glucopyranoside (PNPG, Sigma-Aldrich, Germany) at 40°C and pH 7.0 (50 mM potassium phosphate buffer). The optimum pH and temperature on β-glucosidase activity were measured over a range of pH from 3.0 to 9.0 and temperature from 25°C to 70°C, respectively. β-Glucosidase activity of the supernatant was determined according to the method [11].

### Conversion of Ginsenoside Rb_1_ by Supernatant of *D. anomala* YAE-1

Selected colonies were inoculated in YM broth (pH 4.0) (HiMedia Laboratories) for 3 days at 30°C. Supernatant activities were tested for hydrolysis of the major ginsenoside Rb_1_ under optimum condition (40°C, pH 5.0) of β-glucosidase. The reaction condition was cultured at 40°C for 48 hours. Conversion of ginsenoside Rb_1_ was checked by TLC and HPLC analysis [12].

### Fermentation of Ginseng Root by *D. anomala* YAE-1

Ginseng roots were sliced and sterilized at 75°C for 10 min. A 20% (w/v) ginseng root solution was incubated with 3% (v/v) of yeast strain YAE1 at 30°C for 7 days. The fermentation characteristics of ginseng root by the strain YAE-1 were analyzed by TLC and HPLC, and viable cell counts were performed according to the method [12].

### Analysis of Ginsenoside Hydrolysates

Hydrolyzed ginsenosides obtained from filtrate samples showing β-glucosidase activity were analyzed using the reversed-phase HPLC system Shimadzu LC-6AD (Shimadzu, Japan) and an ACE-5-C18 column (4.6 × 250 mm) equilibrated with solvent A (H2O). HPLC analysis was conducted according to the method [12].

### Statistical Analysis

All analyses were repeated at least 3 times and are expressed as means ± standard deviation (SD) [12].

## Results and Discussion

### Isolation and Screening of the Strain Exhibiting β-Glucosidase Activity

**Fig. 1:**
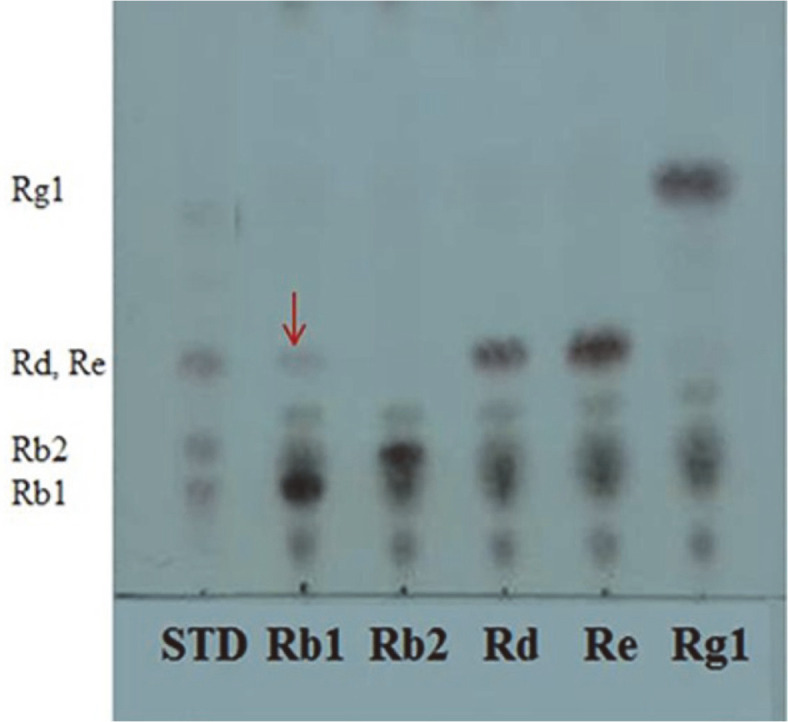
TLC analysis of ginsenoside Rb1, Rb2, Rd, Re and Rg1 fermented by *D. anomala* YAE-1 at 30°C for 48 h. TLC was performed on Silica gel 60 F_254_ plates. A solvent mixture of chloroform: methanol: water (65:35:10, v/v/v, lower phase) was used as the developing solvent.

Samples of the Mongolian traditional fermented dairy product airag were collected from 5 different farms. The respective isolated strains were: Airag A (strain YAA1-YAA7), Airag B (strain YAB1-YAB8), Airag C (strain YAC1-YAC6), Airag D (strain YAD1-YAD8), Airag E (strain YAE1-YAE8), and they were examined for β-glucosidase activity. Fifteen strains showed esculin-positive reaction. The YAE-1 strain was found to be the strongest esculin positive. When the major ginsenosides Rb_1_, Rb_2_, Rd, Re, and Rg1 were hydrolyzed by the strains, strain YAE-1 can hydrolyze ginsenoside Rb_1_ converted to Rd in minor quantity, while no conversions occurred for ginsenosides Rb_2_, Rd, Re and Rg1 (Fig. 1). β-Glucosidase is mainly a hydrolyzing enzyme for conversion of protopanaxadiol ginsenosides in ginseng. Many types of microorganisms with β-glucosidase activity have been used to hydrolyze ginsenosides Rb_1_, Rb_2_, Rc, and Rd to minor ginsenoside F_2_ and compound K [15-18].

*Penicillium dipodomyicola* strain isolated from the soil of wild ginseng also biotransformed major ginsenosides into compound K. The optimum transforming conditions for this fungus are 40°C, medium pH of 4.0 - 6.0 and incubation time of 7 days [15]. *Aspergillus usamii* KCTC6954 converted ginsenoside Rb_1_ to compound K and the incubation time was 15 days. The optimum temperature and pH of β-glucosidase produced by this mold is 60°C and 6.0 [16]. On the other hand, *Candida allociferrii* JNO301 isolated from meju (fermented soybean) converted ginsenosides of red ginseng extract, and the optimum temperature and pH were 20-30°C and 5-8, respectively. *C. allociferrii* JNO301 converted ginsenoside Rb_1_ to Rd → F2, Rb2 → Compound O, Rc → Mc1, Rf → Rh_1_ [17]. The β-glucosidase purified from the tomato pathogen *Cladosporium fulvum* was hydrolyzed with Rb_1_ to Rd. The enzyme has an optimal pH of 5.5 and an optimal temperature of 45°C [18].

**Fig. 2:**
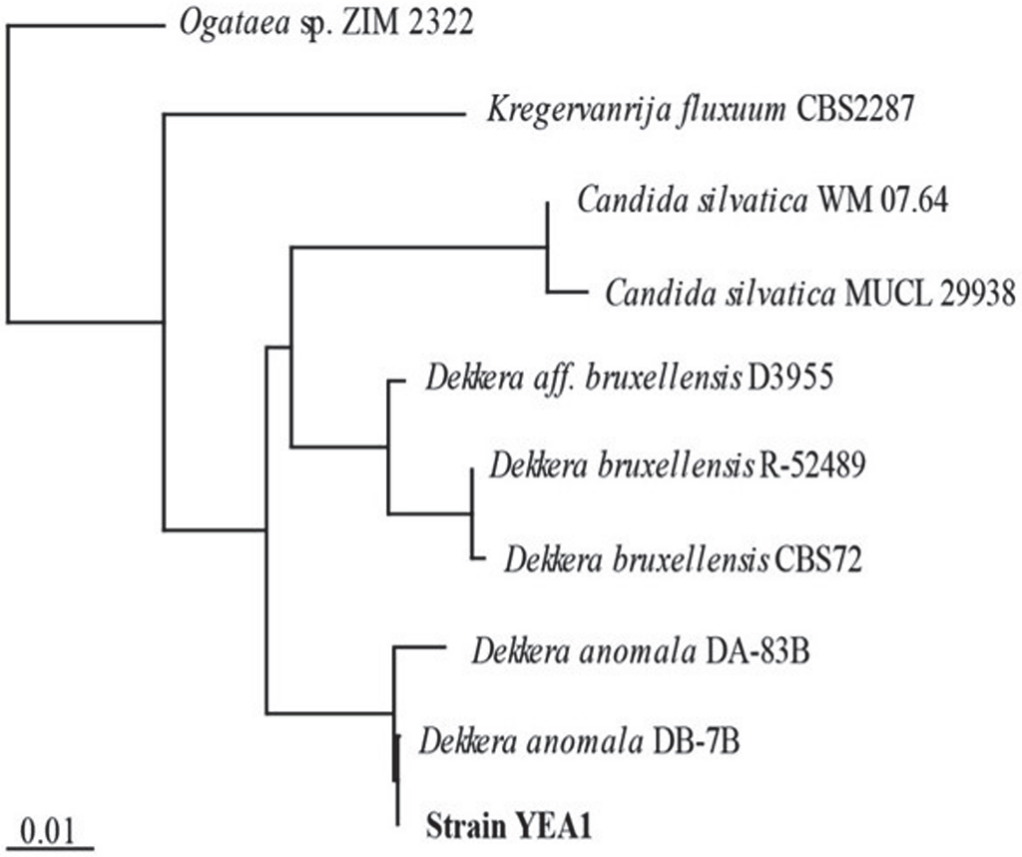
Phylogenetic tree based on 18S rRNA sequences showing the position of yeast strain YAE-1. Scale length is 0.01.

Strain YAE-1 was selected for DNA analysis. The PCR produced 18S rRNA sequences of the strain YAE-1. The sequence of the strain was compared using the NCBI database, and it was found to be 99.9% homologous with *D. anomala* DB-7B (Fig. 2). The isolated YAE-1 strain was thus named as *D. anomala* YAE-1.

### Optimum Temperature and pH for Growth of *D. anomala* YAE-1

**Fig. 3:**
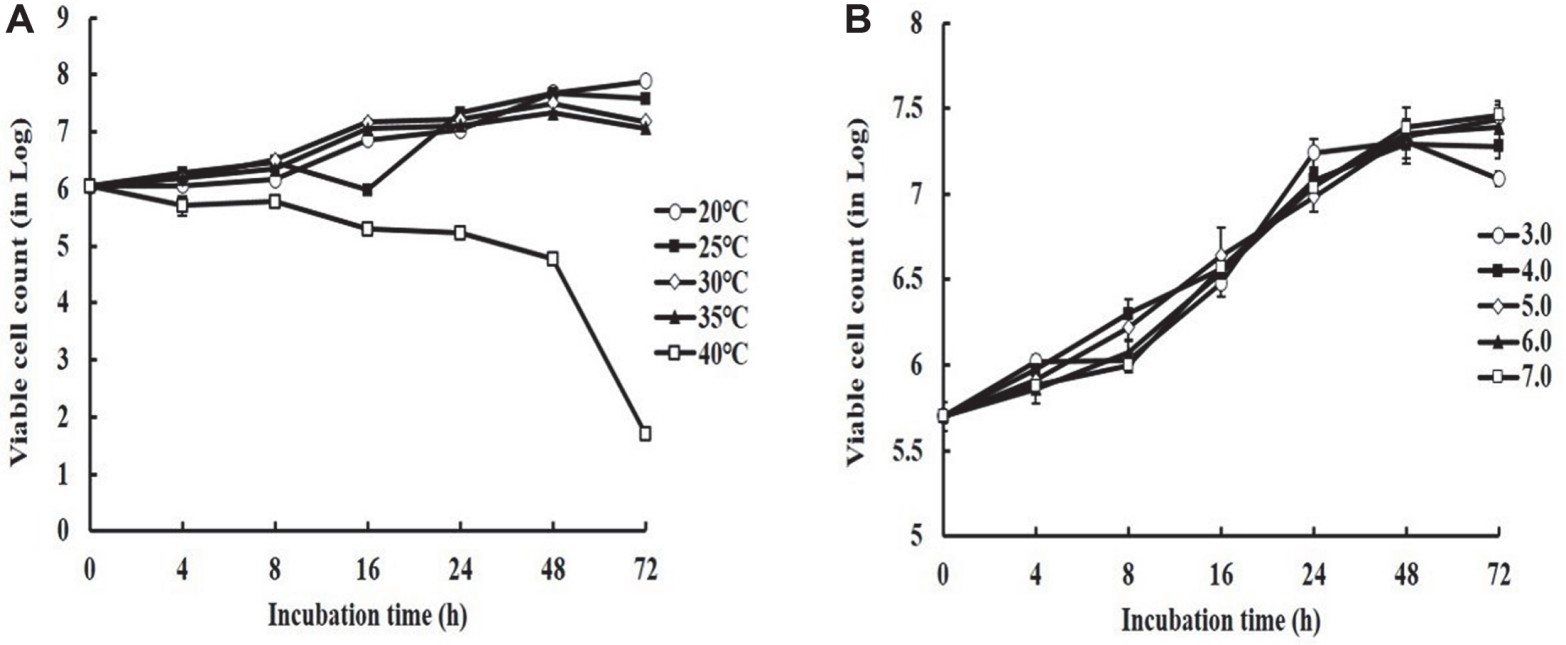
Effect of temperature and pH on growth of *D. anomala* YAE-1. A: Temperature, B: pH.

The effect of temperature and pH on growth of *D. anomala* YAE-1 is shown in Fig. 3. The number of viable cells started increasing at 1.12 × 10^6^ CFU/ml, but at 40°C, the number of viable cells rapidly decreased to 6.0 × 10^4^ CFU/ml after 48 h incubation, and 5.0 × 101 CFU/ml after 72 h incubation at 40°C. However, the viable cell count was highest at 4.78 × 10^7^ CFU/ml after 48 h incubation at 25-30°C. The optimum temperature for growth of *D. anomala* YAE-1 was found to be 25°C and 30°C, where its growth rate increased at these two temperatures compared to other temperature treatments during the incubation (Fig. 3A).

By changing the pH of the medium, *D. anomala* YAE-1 grew well at pH 4.0 during the beginning stage of incubation, but also grew well at pH 7.0 during 48 and 72 h incubation (Fig. 3B). Optimum growth temperature and pH of *D. anomala* generally were between 25°C to 30°C, and pH 7.0, respectively.

### Enzymatic Activity and Carbohydrate Utilization of *D. anomala* YAE-1

Enzymatic activities of *D. anomala* YAE-1 strain exerted higher β–glucosidase activity (≥ 30 nmol) than α-galactosidase (≥ 5 nmol) and α–glucosidase (0 nmol). On the other hand, fungi (*P. dipodomyicola* and *A. usamii* KCTC6954), bacteria (*Cl. fulvum*) and yeast (*C. allociferrii* JNO301) had β–glucosidase activity [15-18]. *D. anomala* YAE-1 fully consumed carbohydrates such as glucose, D-galactose, N-acetyl-D-glucosamine, D-cellulose, D-lactose, while glycerol and raffinose were consumed 90% and 50%, respectively. *C. allociferrii* JNO301 can utilize carbohydrates such as glucose, galactose, N-acetyl-D-glucosamine, glycerol and raffinose, but lactose is not utilized [17].

### Optimum Temperature and pH of β-Glucosidase Activity in Supernatant

**Fig. 4:**
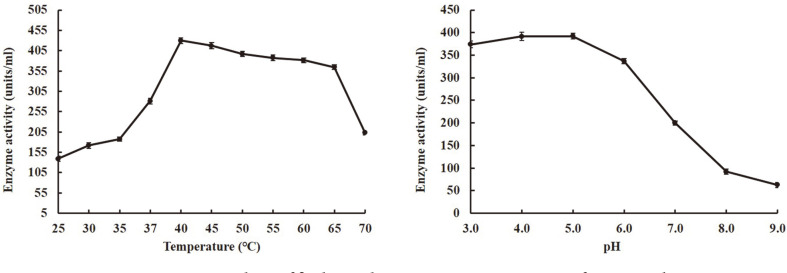
Optimum temperature and pH of β-glucosidase activity in supernatant of *D. anomala* YAE-1.

The β-glucosidase of the supernatant exhibited maximum enzyme activity at 40°C (Fig. 4), but gradually decreased activity at 45 to 65°C, and finally showed significantly decreased activity above 65°C. The optimum pH for the activity occurred around 4.0 to 5.0 at 30°C. The enzyme activity was rapidly decreased above pH 5. Enzyme maximal activity was at pH 5.0 (Fig. 4). On the other hand, the optimum pH and temperature of β-glucosidase isolated from *Cl. fulvum* was 5.5 and 45°C, respectively [18]. Furthermore, β-glucosidase produced by *P. dipodomyicola* isolated from soil of wild ginseng best converted ginsenoside Rb_1_ to minor ginsenosides under a pH value from 4.0 to 6.0 and the best fermentation temperature was 40°C [15]. Thus, the optimum conditions for β-glucosidase activity are considered to be in the range of pH 5.0-5.5 at the temperature of 40-45°C.

### Biotransformation of Ginsenoside Rb_1_ to Rd by *D. anomala* YAE-1

**Fig. 5:**
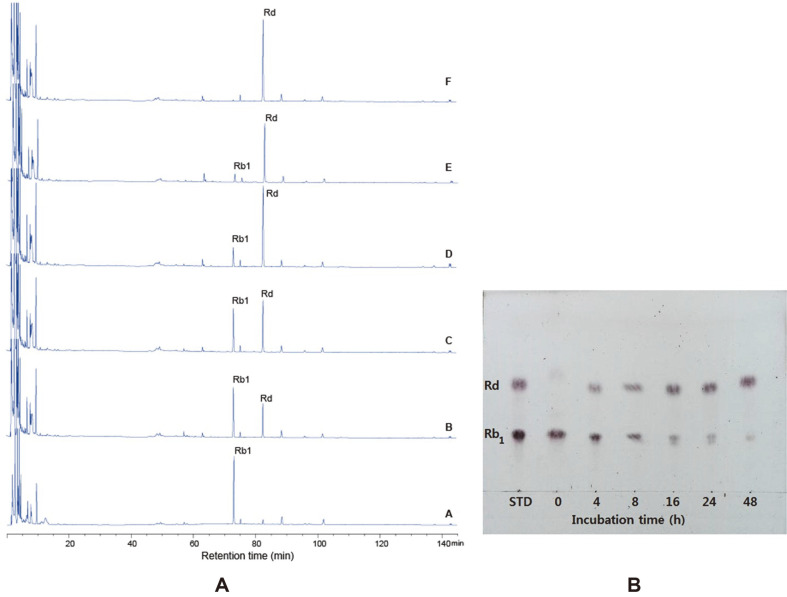
Biotransformation of ginsenoside Rb_1_ hydrolyzed by supernatant of *D. anomala* YAE-1 for 48 h. A: HPLC analysis, 0 h, B-4 h, C-8 h, D-16 h, E-24 h, F-48 h; TLC analysis.

D. anomala YAE-1 exhibiting β-glucosidase activity was incubated in YM broth (pH 4.0) for 48 h at optimum condition of 30°C. Hydrolysis of the major ginsenoside Rb_1_ was tested under optimum condition for β-glucosidase (40°C, pH 5.0). After 48 h, 3.095 mg/ml of ginsenoside Rb_1_ was fully hydrolyzed by supernatant with β-glucosidase activity, and released 3.0 mg/ml of ginsenoside Rd (Fig. 5), (Table 1A). Our results showed that Rd was the main ginsenoside in the final fermentation product of ginsenoside Rb_1_. Enzymatic hydrolysis made it possible to produce minor ginsenosides. The biological activities of ginsenosides increase according to their molecular mass. [19]. Many reports have been conducted on the conversion of the ginsenoside Rb_1_ into Rd during fermentation [13, 20, 21]. Our results on the biotransformation of ginsenoside Rb_1_ by *D. anomala* YAE-1 revealed that there were some similarities with the previous studies conducted with different types of strains. Also, most of the microorganisms used for the transformation of ginsenoside do not meet food-grade standards and researchers were searching for microorganisms that are safe for use in various foods [22]. Most microorganisms with β-glucosidase activity cannot hydrolyze major ginsenosides and β-glucosidase hydrolyzing ginsenosides very rarely occur among foodborne microorganisms [12]. Jo et al. [17] reported that after 48 h at 60°C, β-glucosidase enzymes of 3- and 6-day cultures of *A. usamii* KCTC6954 were unable to convert the ginsenoside Rb_1_ and enzymes of 9-, 12- and 15-day cultures fully converted ginsenoside Rb_1_ to ginsenoside Rd, F_2_ and compound K. Moreover, Lunpeng et al. [15] observed that after 48 h, β-glucosidase produced by *P. dipodomyicola* isolated from soil of wild ginseng completely converted ginsenoside Rb_1_ to ginsenoside Rd, and subsequently, ginsenoside Rd completely converted to compound K after 7 days. In addition, β-glucosidase isolated from *P. dipodomyicola* was able to convert ginsenoside Rb_2_ to compound Y; ginsenoside Rc to compound Mc; and ginsenoside Rd to compound K.

### Characteristics of Ginseng Root Fermented by *D. anomala* YAE-1

**Fig. 6:**
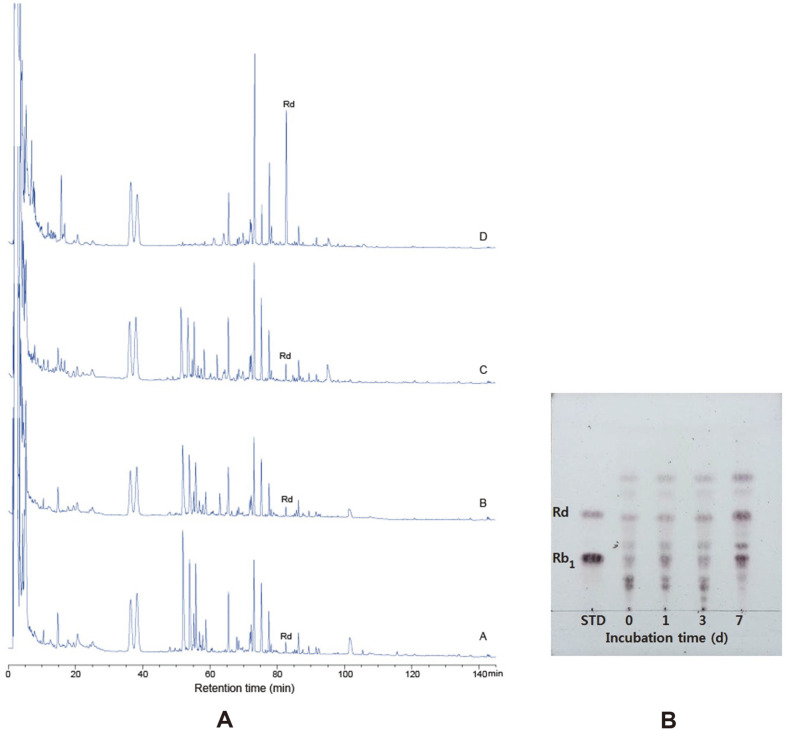
Biotransformation of ginsenoside of fermented ginseng root by *D. anomala* YAE-1 during 0, 1, 3, 7 days. A: HPLC analysis, A-0 day, B-1 days, C-3 days, D-7 days. B: TLC analysis.

D. anomala YAE-1 hydrolyzed ginsenoside Rb_1_ to ginsenoside Rd in 20% fermented ginseng root. The peak size of ginsenoside Rb_1_ was small and that of ginsenoside Rd was significantly large after 7 days, as revealed in HPLC chromatograms (Fig. 6). Furthermore, the quantity of ginsenoside Rd was elevated from 0 to 1.404 mg/ml at the same period (Table 1B). In fermented ginseng roots, ginsenoside Rb_1_ was converted to Rd, reducing ginsenoside Rb_1_ and increasing Rd. The factor that absolutely affects ginsenoside conversion is fermentation time. Rd was increased as the main ginsenoside in the final fermentation product of ginseng roots, confirming that Rb_1_ was decreased continuously during the 7-day fermentation. The quantity of minor ginsenoside Rd was elevated from 0 to 0.060 ± 0.011 mg/ml at 7 days in *Paenibacillus* sp. MBT213, which converts ginsenoside Rb_1_ into ginsenoside Rd from *Panax ginseng* [12]. The ginsenoside conversion by *Paenibacillus* sp. MBT213 was absolutely affected by fermentation time as was the ginsenoside conversion by *D. anomala* YAE-1. The initial viable cell count of *D. anomala* YAE-1 was 4.85 log CFU/ml, and maximum cell count was 7.39 log CFU/ml after 7 days (Table 1C). *D. anomala* YAE-1 has strong β-glucosidase activity, which can be used to convert major ginsenoside Rb_1_ to Rd during the fermentation of ginseng. It is also believed that the converted ginsenoside Rd has the potential to be used in a variety of health functional foods and pharmaceutical products.

## Figures and Tables

**Table 1:** Quantities of biotransformation of ginsenoside Rb_1_ to Rd and viable cell counts of 20% fermented ginseng by *D. anomala* YAE-1.

		Incubation time (h)					

		0	4	8	16	24	48

A	Rb_1_ (mg/ml)	3.095±0.012^*a^	2.331±0.02^b^	1.946±0.029^c^	0.962±0.014^d^	0.698±0.010^e^	0.00^f^
Rd (mg/ml)	0.055±0.007^f^	0.669±0.069^e^	1.044±0.034^d^	2.038±0.046^c^	2.302±0.014^b^	3.0±0.096^a^	


				Incubation time (day)			

				0	1	3	7

B	Rb_1_ (mg/ml)			0.765±0.026^d^	1.1751±0.11^c^	1.7384±0.0446^b^	4.8076±0.0873^a^
	Rd (mg/ml)			0.003±0.0011^d^	0.0287±0.0016^c^	0.0737±0.0039^b^	1.4041±0.03130^a^
C	*D. anomala* YAE-1 (Log CFU/ml)			7.2×10^4^	1×10^6^	1×10^7^	2.5×10^7^

^*^Means ± SD (*n* = 3)

^a-f^Means with different superscript letters among the same row are significantly different at *p* < 0.05.

A) Quantities of biotransformation of ginsenoside Rb_1_ by supernatant of *D. anomala* YAE-1 during 48 h. The quantities of ginsenoside Rb_1_ and Rd were determined by HPLC.

B) Quantities of biotransformation of ginsenoside Rb_1_ and Rd in ginseng root by *D. anomala* YAE-1 during 7 days. The amount of ginsenoside Rb_1_ and Rd were determined by HPLC.

C) Viable cell counts of *D. anomala* YAE-1 in 20% fermented ginseng root.
